# Antibodies directed against endogenous and exogenous citrullinated antigens pre-date the onset of rheumatoid arthritis

**DOI:** 10.1186/s13075-016-1031-0

**Published:** 2016-06-03

**Authors:** Linda Johansson, Federico Pratesi, Mikael Brink, Lisbeth Ärlestig, Claudia D’Amato, Debora Bartaloni, Paola Migliorini, Solbritt Rantapää-Dahlqvist

**Affiliations:** Clinical Immunology Unit, Department of Clinical and Experimental Medicine, University of Pisa, Pisa, Italy; Department of Public Health and Clinical Medicine/Rheumatology, Umeå University, Umeå, Sweden

## Abstract

**Background:**

Anti-citrullinated-peptide antibodies (ACPA) have been detected in individuals with developing rheumatoid arthritis (RA) before the onset of symptom, with an initially limited spectrum of reactivities that gradually broadens. The aim was to analyze the evolution of ACPA response pre-dating symptom onset, using four selected citrullinated exogenous and endogenous antigens.

**Methods:**

A cohort of 521 individuals sampled before symptoms of RA appeared and 272 population controls were identified from the Biobank of Northern Sweden; 241 samples from patients with early RA were also collected. ACPA were detected by ELISA on viral citrullinated peptides (VCP) derived from Epstein-Barr-virus nuclear antigen (EBNA)1 and EBNA2 (VCP1 and VCP2) and histone-4-derived citrullinated peptides (HCP1 and HCP2).

**Results:**

In pre-symptomatic individuals vs. patients with early RA, anti-VCP1 antibodies were detected in 10.4 % vs. 36.1 %, anti-VCP2 in 17.1 % vs. 52.3 %, anti-HCP1 in 10.2 % vs. 37.3 %, and anti-HCP2 in 16.3 % vs. 48.5 %, respectively. Anti-VCP and anti-HCP concentrations were significantly increased in pre-symptomatic individuals vs. controls (*p* < 0.001) and were increased approaching symptom onset. Anti-VCP and anti-HCP appeared simultaneously (median (IQR) 5.3 (6) years before symptom onset) and in combination yielded a high-risk ratio for disease development (OR = 8.0–18.9). Anti-VCP2 and anti-HCP2 antibodies were associated with HLA-DRB1*0401 in pre-symptomatic individuals. Three peptidylarginine deiminase (PAD)I3/PADI4 single nucleotide polymorphisms (SNPs) were significantly associated with anti-HCP1.

**Conclusions:**

Anti-VCP and anti-HCP antibodies pre-date symptom onset and predict disease development, but no hierarchy of citrullinated epitopes can be identified. These results suggest that no inciting citrullinated antigen so far described is common to all patients with RA. The association between PADI3/PADI4 polymorphism and anti-HCP1 antibodies suggests a novel link between deimination and production of ACPA.

**Electronic supplementary material:**

The online version of this article (doi:10.1186/s13075-016-1031-0) contains supplementary material, which is available to authorized users.

## Background

Rheumatoid arthritis, a chronic inflammatory autoimmune disease, is a complex and multifactorial disorder influenced by both genetic and environmental factors [1]. A distinctive feature of RA is the presence of anti-citrullinate-peptide antibodies (ACPA), i.e*.*, autoantibodies directed against peptides/proteins in which arginine residues have been post-translationally modified into citrulline by peptidilarginine deiminase [2]. ACPA may recognize numerous citrullinated antigens: filaggrin, alpha and beta fibrin/fibrinogen, alpha-enolase, vimentin, histones, and Epstein-Barr virus (EBV)-derived proteins [3, 4]. Because of their strict disease specificity, ACPA have recently been included in the American College of Rheumatologists/European League Against Rheumatism (ACR/EULAR) criteria for diagnosis of RA [5].

ACPA appear very early in the course of the disease, even years before disease manifestations, with an increase in concentrations approaching disease onset [6, 7]. During pre-clinical phases, ACPA recognize a limited number of citrullinated antigens and display low affinity for their antigenic targets [8]. Epitope spreading towards other citrullinated epitopes and avidity maturation occur, and a full repertoire of high-affinity ACPA is established before the onset of the disease and is associated with the disease course [8–10].

The events that take place during the pre-clinical stage and finally lead to the production of high-affinity polyclonal ACPA are not yet elucidated. A current hypothesis is that inflammatory/infectious stimuli induce deimination of exogenous or endogenous antigens, leading to the expansion and differentiation of ACPA-producing B cells, an event occurring mainly in ectopic lymphoid structures (ELS) [11]. The trigger of infectious agents is a possible explanation. EBV, proposed as an inducing agent in RA on the basis of epidemiological and serological data, is an ideal candidate and its presence in synovial ELS further supports its possible role [11, 12]. Alternatively, B-cell differentiation may be induced by the release of citrullinated antigens in a molecular milieu abundant with danger signals. The recent discovery of neutrophil extracellular traps (NETs) as a source of citrullinated antigens, and namely citrullinated histones H3 and H4 [13, 14], intimately associated with pro-inflammatory signals such as LL-37 [15], may be consistent with this hypothesis. In fact, synovial fluid neutrophils have enhanced NETosis in RA, exposing citrullinated antigens that may fuel the production of ACPA [16, 17]. These observations led to the hypothesis that aberrant NETosis is a triggering event in RA, as suggested for other autoimmune diseases. We have previously described deiminated sequences contained in EBV-derived proteins EBNA1 and EBNA2 as specific targets of ACPA, [18, 19] and that sera from patients with RA also react with deiminated H4 released during NETosis [14].

We took advantage to focus on these fully characterized ACPA targets, corresponding to exogenous antigens (i.e., viral citrullinated peptide (VCP)1 derived from EBNA1, VCP2 from EBNA2) or endogenous antigens (histone citrullinated peptide (HCP)1 and HCP2 from histone H4), to analyse a cohort of individuals without joint symptoms who have subsequently developed RA. The aim of the present work was to test the hypothesis that there is a hierarchy of citrullinated epitopes between exogenous and endogenous antigens in the build-up of the ACPA response in patients with RA.

## Methods

### Study population

A nested case-control study was performed based on individuals included in population-based survey cohorts within the Medical Biobank of Northern Sweden. The criteria concerning the recruitment, blood sampling and storage conditions (−80 ˚C) have been described previously [6]. The register of patients attending the Department of Rheumatology, University Hospital of Umeå, and who fulfilled the 1987 classification criteria for RA [20], were co-analysed with the registers of the Medical Biobank cohorts. The study included 531 individuals (153 men and 378 women) referred to as pre-symptomatic individuals, all of whom had donated at least one sample before the disease onset of RA, and 277 population-based controls (107 men and 170 women) randomly selected from the same cohorts within the Medical Biobank. Ten samples from identified pre-symptomatic individuals and five samples from the controls were no longer available. Thus, 521 samples (from 150 men and 371 women) from the pre-symptomatic individuals and 272 samples (from 105 men/167 women) from the controls were analysed. The median (IQR) time pre-dating the onset of symptoms of RA was 5.3 (6.0) years. A separate group of 241 patients (74 men and 67 women) who had donated blood samples at the time of RA diagnosis were included, all of whom had also donated a sample pre-dating symptom onset. All of the individuals were classified as either being a “never-smoker” or “ever a smoker” (including former or current smokers). Descriptive data for the pre-symptomatic individuals, patients with RA and controls are presented in Table 1.

**Table 1:** Descriptive data for the pre-symptomatic individuals, patients with rheumatoid arthritis (RA) and the controls

	Pre-symptomatic individuals (n = 521)	Patients with RA (n = 241)	Controls (n = 272)
Female sex, n (%)	371 (71.2)	167 (69.3)	167 (61.4)
Age, mean (SD)^a^, years	52.4 (9.4)	60.1 (9.7)	51.8 (9.5)
Ever-smoker, n (%)	327/520 (62.9)	162/240 (67.5)	110/266 (41.4)
HLA-SE carrier^b^, n (%)	249/392 (63.5)	147/218 (67.4)	-
PTPN22 1858 T-variant carriage, n (%)	92/262 (35.1)	56/192 (29.2)	-
Anti-CCP2 antibody, n (%)	190/518 (36.7)	189/241 (78.4)	7/272 (2.6)

^a^Mean age (standard deviation) calculated for all pre-symptomatic individuals (n = 521) and patients with RA (n = 241). ^b^HLA-DRB1 shared epitope (SE) defined as 0101/0401/0404/0405/0408

Radiographs of the hands and feet, available for 177 patients with RA at baseline and after 24 months, were graded according to the Larsen score [21]. The smallest detectable difference in the score was calculated to be 4 [22].

### Antigenic peptides

Peptides used in this study were synthesized as multiple antigen peptides as follows: VCP1 (CitEBNA1_35-58_):(GGDNHG**Cit**G**Cit**G**Cit**G**Cit**G**Cit**GGG**Cit**PGAPG)_4_K_2_KßA; VCP2 (CitEBNA2_338-358_):(GQS**Cit**GQS**Cit**G**Cit**G**Cit**G**Cit**G**Cit**G**Cit**GKG)_4_K_2_KßA; HCP1 (CitH4_14-34_):(GAK**Cit**H**Cit**KVL**Cit**DNIQGITKPAI)_4_K_2_KßA; and HCP2 (CitH4_31-50_):(KPAI**CitCit**LA**CitCit**GGVK**Cit**ISGLI) _4_K_2_KßA.

### Determination of anti-VCP and anti-HCP antibodies

Anti-VCP and anti-HCP antibodies were determined by ELISA as previously described [14, 18, 19]. In each ELISA a 7-point titration curve of a pool of ACPA+ RA plasma with twofold dilution was included, setting the highest concentration of the ACPA+ pool at 100 arbitrary units (AU)/ml. Receiver operating characteristic (ROC) curves were used to define the cutoff values for antibody positivity. The cutoff for anti-VCP1 IgG positivity was set at 32.89 AU/ml), anti-VCP2 IgG at 9.09 AU/ml, anti-HCP1 IgG at 19.51 AU/ml and anti-HCP2 IgG at 11.64 AU/ml, corresponding to specificity of 98.2 %.

### Analyses of anti-CCP2 antibodies and ACPA

Plasma were analysed using a microarray based on the ImmunoCAP ISAC system (PhaDia) described previously for IgG ACPA specificity [8]. For this study only the three antibodies with the highest frequencies analysed on the same cohort of pre-symptomatic individuals as presented in the previously mentioned study were selected; α-enolase (CEP-1/Eno5-21, citrullinated at 9 and 15), fibrinogenβ36-52 (Fibβ36-52, citrullinated at 44) and filaggrin (CCP-1/Fil307-324, citrullinated at 312). The cutoff values for ACPA were defined according to ROC-curve analysis [8]. The presence of anti-citrullinated protein antibodies (anti-CCP2), was also assessed with a cutoff of 25 AU/ml according to the manufacturer’s protocol (Euro Diagnostica, Malmö, Sweden).

### Genotyping

For the genotyping of *HLA-DRB1*, shared epitope (SE) defined as HLA-DRB1*0401/0404/0405/0408/0101, polymerase chain reaction sequence-specific primers were used, from a DR low-resolution kit and DRB1*04 and *01 subtyping kit (Dynal, Oslo, Norway) [23]. Data on gene polymorphisms were extracted from Immunochip analysis (SNP&SEQ Technology Platform Uppsala, Sweden) covering 314 single nucleotide polymorphisms (SNPs) for *PADI* gene loci [24]. Information about the protein tyrosine phosphatase, non-receptor type 22 (*PTPN22*) C1858T was also extracted from the Immunochip analysis.

### Statistics

Continuous data were compared using the Mann-Whitney *U* test, and the Kruskal-Wallis test for more than two groups. Categorical data were analysed using the chi-square (X^2^) test or Fisher’s exact test, and Spearman’s rank correlation test (*r*_s_). The frequency distribution was compared using Kendall’s-tau test. Logistic regression analysis was used to identify associations between antibodies and disease development and is presented as the odds ratio (OR) with 95 % confidence interval (CI). Univariate analysis of variance was used for analysing relationships between antibodies and continuous data. All of the adjustments were based on the results from previously performed studies, and *p* values ≤0.05 were considered significant. All of the statistical analyses were performed using SPSS 23.0 software (Chicago, IL, USA). Genetic analyses of SNPs in relation to concentration were performed using PLINK (1.07) [25] with Bonferroni corrections and Haploview (4.2), a program used for haplotype analysis (http://www.broad.mit.edu/mpg/haploview).

## Results

### Concentrations of anti-VCP1, anti-VCP2, anti-HCP1 and anti-HCP2 IgG in pre-symptomatic individuals, patients with RA and controls

**Fig. 1:**
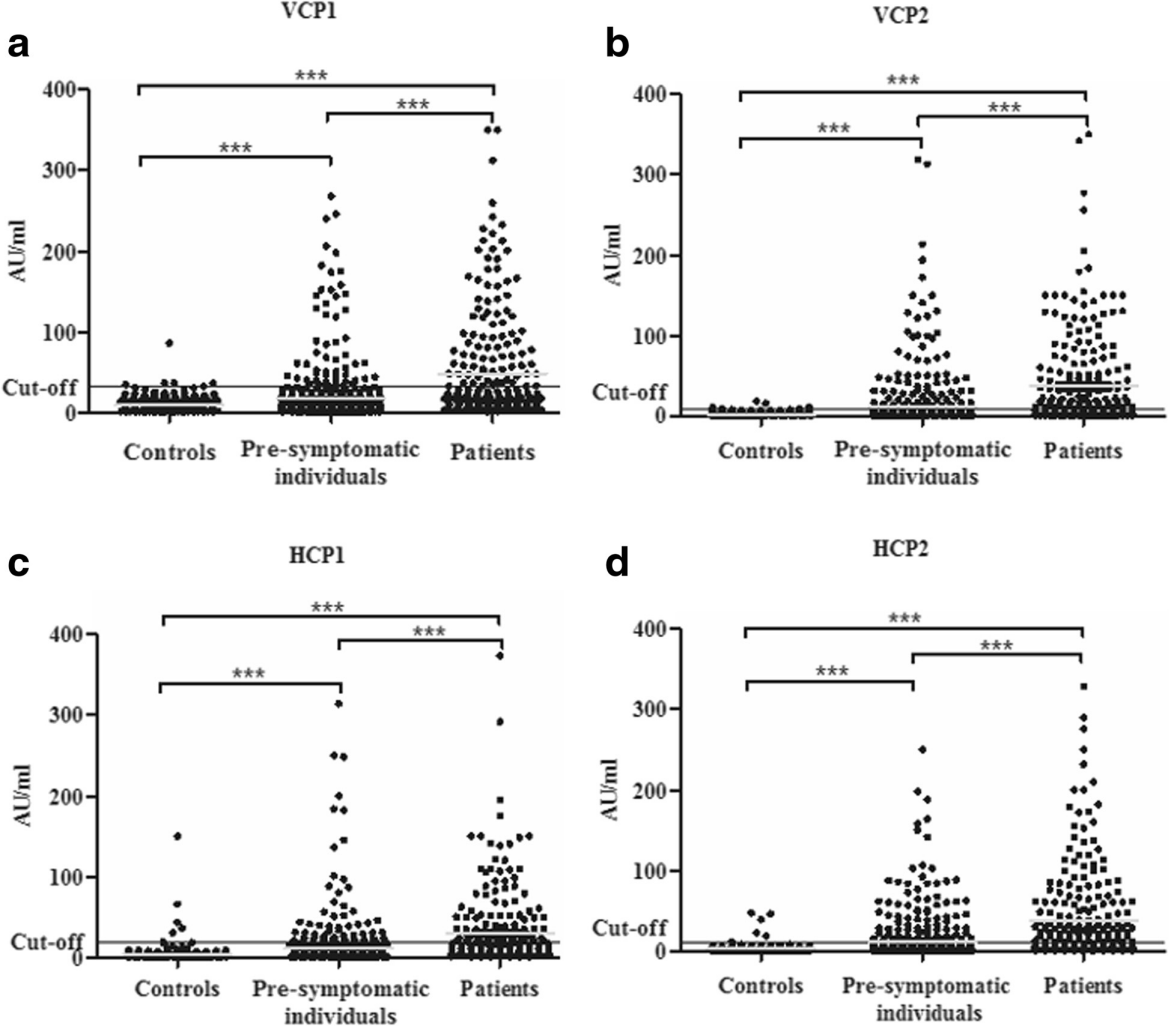
**a** Concentrations of antibodies against viral citrullinated peptides derived from Epstein-Barr-virus derived protein (*VCP1*) in controls, pre-symptomatic individuals and patients with rheumatoid arthritis (RA). *Grey line* indicates the mean of the concentrations; ***p < 0.001. **b** Concentrations of anti-VCP2 antibodies in controls, pre-symptomatic individuals and patients with RA. *Grey line* indicates the mean of the concentrations; ***p < 0.001. **c** Concentrations of antibodies against histone 4-derived citrullinated peptides (*HCP1*) in controls, pre-symptomatic individuals and patients with RA. *Grey line* indicates the mean of the concentrations; ***p < 0.001. **d** Concentrations of anti-HCP2 antibodies in controls, pre-symptomatic individuals and patients with RA. *Grey line* indicates the mean of the concentrations; ***p < 0.001. *AU* arbitrary units

The concentrations of anti-VCP1, anti-VCP2, anti-HCP1 and anti-HCP2 IgG were all significantly increased in the pre-symptomatic individuals and patients with RA compared with controls (*p* < 0.001 for all eight analyses; cases vs. controls) (Fig. 1a–d). The concentrations were (mean ± SEM) 17.9 ± 1.5 AU/ml, 11.5 ± 1.4 AU/ml, 10.8 ± 1.2 AU/ml, and 11.3 ± 1.2 AU/ml in pre-symptomatic individuals, and 47.9 ± 4.2 AU/ml, 36.9 ± 3.7 AU/ml, 29.2 ± 3.0 AU/ml, and 38.0 ± 3.7 AU/ml in the patients with RA, respectively, vs. 9.1 ± 0.5 AU/ml, 2.4 ± 0.14 AU/ml, 4.4 ± 0.6 AU/ml, and 2.9 ± 0.3 AU/ml in the controls. The concentrations of all four antibodies were significantly increased in patients with RA compared with the pre-symptomatic individuals both at a group level and on an individual level (*p* < 0.001 for all analyses) (Fig. 1a–d).

### Frequency and combinations of positivity of anti-VCP1, anti-VCP2, anti-HCP1 and anti-HCP2 IgG in pre-symptomatic individuals and patients with RA in relation to controls

**Fig. 2:**
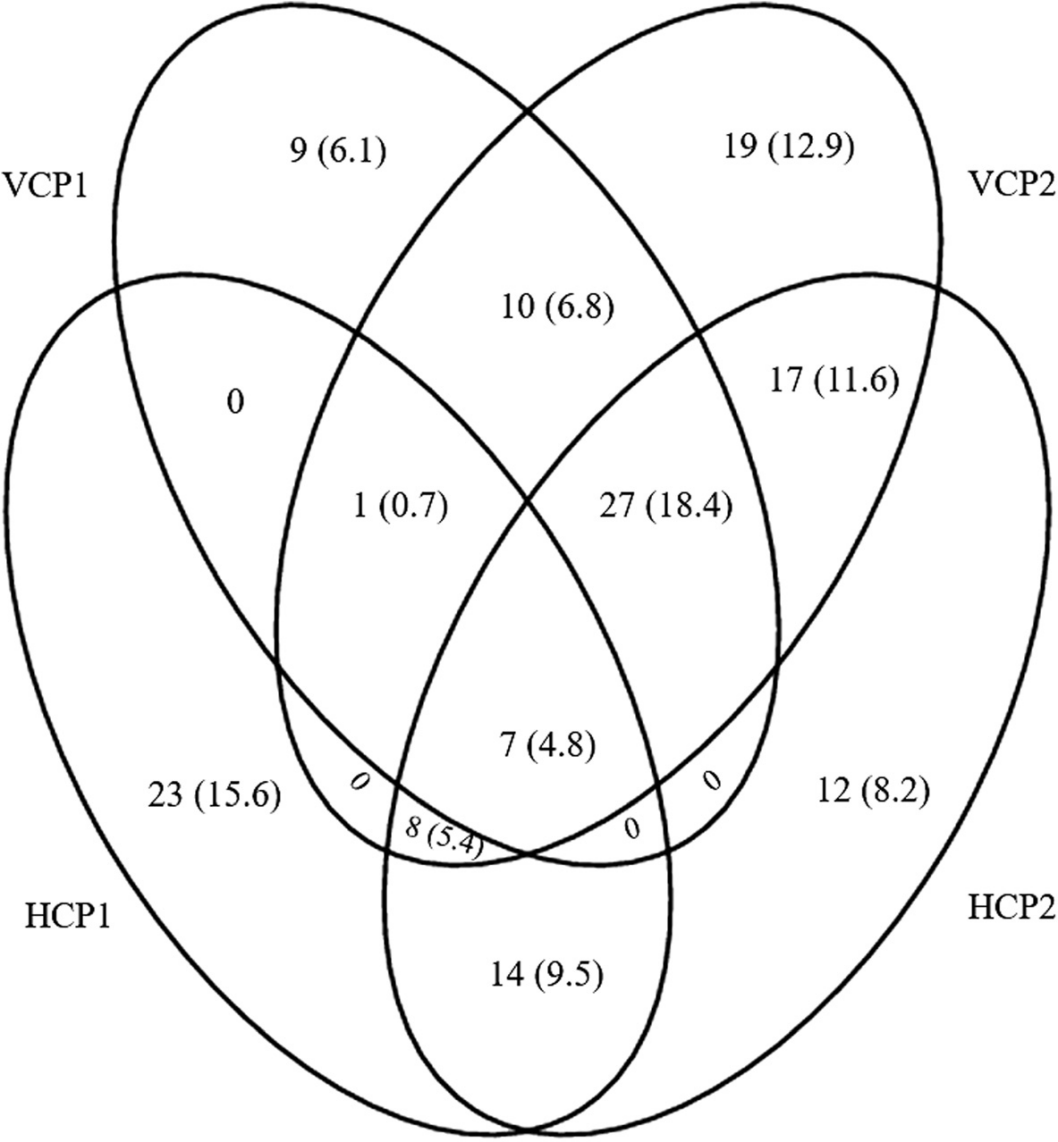
Combinations of positivity of the antibodies, anti-viral citrullinated peptides derived from Epstein-Barr-virus encoded protein (*VCP1*), anti-VCP2, anti-histone 4-derived citrullinated peptides (*HCP1*) and anti-cyclic citrullinated peptide (*CCP2*) in pre-symptomatic individuals. Results are presented as number and percentage of positive individuals, n (%)

The frequency of the antibodies in pre-symptomatic individuals was highest (17.1 %) for anti-VCP2, followed by anti-HCP2 at 16.3 %. The pattern was similar for the patients with RA with a frequency of 52.3 % for anti-VCP2 antibodies and 48.5 % for anti-HCP2 (Table 2). Overall, samples from 147 pre-symptomatic individuals (28.2 %) were positive for any of the antibodies, and the combination of anti-VCP1, anti-VCP2 and anti-HCP2 positivity was most prevalent, being detected in samples from 27 individuals (18.4 %) (Fig. 2). The most frequent isolated positive antibody was anti-HCP1 (15.6 %) (Fig. 2).

**Table 2:** Frequency and odds ratio for anti-VCP1, anti-VCP2, anti-HCP1 and anti-HCP2 antibodies separately and in combinations, in pre-symptomatic individuals and in patients with rheumatoid arthritis (RA) for disease development

Antibodies	Antibody frequency in pre-symptomatic individuals (n = 521), % (95 % CI)	OR (95 % CI)	Sensitivity in patients with RA (n = 241), % (95 % CI)	OR (95 % CI)	Specificity, % (95 % CI)
VCP1	10.4 (8, 13.3)	6.2 (2.4, 15.6)	36.1 (30.3, 42.3)	30.2 (12.0, 75.9)	98.2 (95.6, 99.3)
VCP2	17.1 (14.1, 20.6)	11.0 (4.4, 27.4)	52.3 (46, 58.5)	58.5 (23.3, 146.8)	98.2 (95.6, 99.3)
HCP1	10.2 (7.9, 13.1)	6.1 (2.4, 15.3)	37.3 (31.5, 43.6)	31.8 (12.7, 80.1)	98.2 (95.6, 99.3)
HCP2	16.3 (13.4, 19.8)	10.4 (4.2, 26.0)	48.5 (42.3, 54.8)	50.4 (20.1, 126.4)	98.2 (95.6, 99.3)
CCP2	36.7 (32.6, 40.9)^a^	21.9 (10.1, 47.4)^a^	78.4 (72.8, 83.1)	137.6 (61.2, 309.6)	97.4 (94.6, 98.8)
VCP1 + VCP2	8.6 (6.4, 11.2)	8.5 (2.6, 27.5)	32.8 (27.7, 39.0)	43.7 (13.6, 140.8)	98.9 (96.6, 99.8)
VCP1 + HCP1	1.5 (0.7, 3.1)	-	17.0 (12.8, 22.3)	1.21 (1.1, 1.3)	100 (98.3, 100)
VCP1 + HCP2	6.5 (4.7, 9)	18.9 (2.6, 139.0)	28.6 (23.3, 34.7)	108.7 (15.0, 790.0)	99.6 (97.7, 100)
VCP2 + HCP1	3.1 (1.9, 5)	-	25.7 (20.6, 31.6)	1.4 (1.3, 1.5)	100 (98.3, 100)
VCP2+ HCP2	11.3 (8.7, 14.2)	17.2 (4.2, 71.1)	40.2 (34.3, 46.6)	90.9 (22.1, 374.2)	99.3 (97.1, 100)
HCP1 + HCP2	5.6 (3.9, 7.9)	8.0 (1.9, 33.6)	26.6 (21.4, 32.5)	48.8 (11.8, 201.9)	99.3 (97.1, 100)
VCP1 + CCP2^a^	8.7 (6.6, 11.5)	25.8 (3.5, 188.1)	35.7 (29.9, 41.9)	150.4 (20.7, 1090.3)	99.6 (97.7, 100)
VCP2 + CCP2^a^	15.8 (12.8, 19.1)	25.2 (6.1, 103.2)	52.3 (46, 58.5)	147.9 (36.0, 608.2)	99.3 (97.1, 100)
HCP1 + CCP2^a^	8.3 (6.2, 11)	24.5 (3.4, 179.2)	35.7 (29.9, 41.9)	150.4 (20.7, 1090.3)	99.6 (97.7, 100)
HCP2 + CCP2^a^	15.4 (12.6, 18.9)	16.4 (5.1, 52.4)	48.5 (42.3, 54.8)	84.6 (26.4, 271.4)	98.9 (96.6, 99.0)

The antibodies are presented for the frequency of positivity, and with odds ratio (OR) and 95 % confidence interval (95 % CI). ^a^Frequency in pre-symptomatic individuals, n = 518. *VCP*, viral citrullinated peptides derived from Epstein-Barr-virus encoded protein; *HCP*, antibodies against histone 4-derived citrullinated peptides, *CCP* cyclic citrullinated peptide

The OR for disease development in pre-symptomatic individuals was higher for anti-VCP2 antibodies followed by anti-HCP2 antibodies, (OR = 11.0, 95 % CI 4.4, 27.4 and OR = 10.4, 95 % CI 4.2, 26.0, respectively) (Table 2). The combination of anti-VCP2+ and anti-HCP2+ antibodies increased the OR for disease development to 17.2 (95 % CI 4.2, 71.1), and anti-VCP1+ HCP2+ antibodies to 18.9 (95 % CI 2.6, 139.0), respectively. The highest ORs were achieved in combinations of anti-VCP1, anti-VCP2 and anti-HCP1, respectively, with anti-CCP2 antibodies (OR = 25.2–25.8), in the pre-symptomatic individuals (Table 2). The most frequent combination of all four antibodies was anti-VCP1+, anti-VCP2+, anti-HCP1– and anti-HCP2+, which yielded a high risk ratio (OR = 18.70, 95 % CI 2.53, 138.47) compared with negativity for all four antibodies as reference (data not shown).

Positivity for three of the antibodies clearly increased the OR for disease development, yielding an OR of 24.93 (95 % CI 3.40, 182.98), compared with those individuals positive for only one of the antibodies, for whom the OR was 6.23 (95 % CI 2.81, 13.83), being negative for all antibodies as the reference (data not shown).

### The anti-VCP1, anti-VCP2, anti-HCP1 and anti-HCP2 antibody concentrations and frequencies over time

**Fig. 3:**
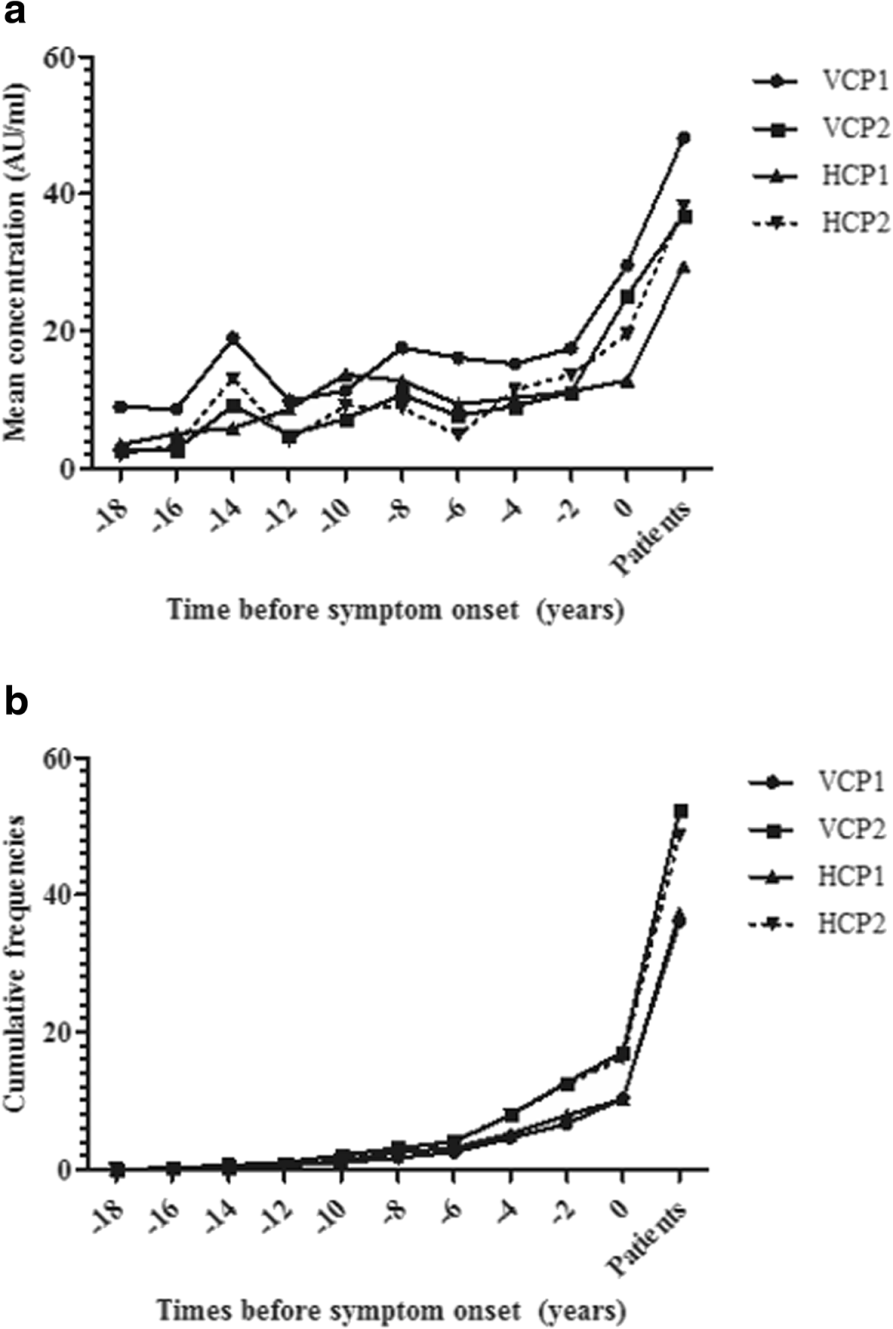
**a** Mean concentration of the four antibodies during the period pre-dating the onset of symptoms up to the onset of rheumatoid arthritis (RA), marked as *0*. **b** Cumulative frequencies of the five antibodies during the period pre-dating the onset of symptoms up to the onset of RA, marked as *0. VCP* viral citrullinated peptide derived from Epstein-Barr-virus encoded protein, *HCP* histone 4-derived citrullinated peptides, *CCP* cyclic citrullinated peptide

The concentrations of anti-VCP1 anti-VCP2, anti-HCP1 and anti-HCP2 antibodies increased gradually over time the closer to onset of symptoms the samples were collected, (*p* < 0.001) (Fig. 3a). Also, the frequency of positivity increased the closer to onset of symptoms the samples were collected for anti-VCP1, anti-VCP2 and anti-HCP2 antibodies (*p* < 0.001 for all three) (Fig. 3b). The number of positive antibodies increased the closer to the onset of symptoms the samples were collected, including all four antibodies (*p* < 0.001).

The time point of the first appearance of the individual ACPA positivity did not differ significantly between the four antibodies (anti-VCP1, anti-VCP2, anti-HCP1, anti-HCP2), or anti-CEP-1, anti-Fibß36-52 or anti-filaggrin antibodies. Positivity appeared first for anti-VCP2 antibodies and the same sample was also positive for anti-filaggrin antibodies (−17.5 years pre symptoms).

### Antibody combinations with anti-CCP2 antibodies and ACPA

**Fig. 4:**
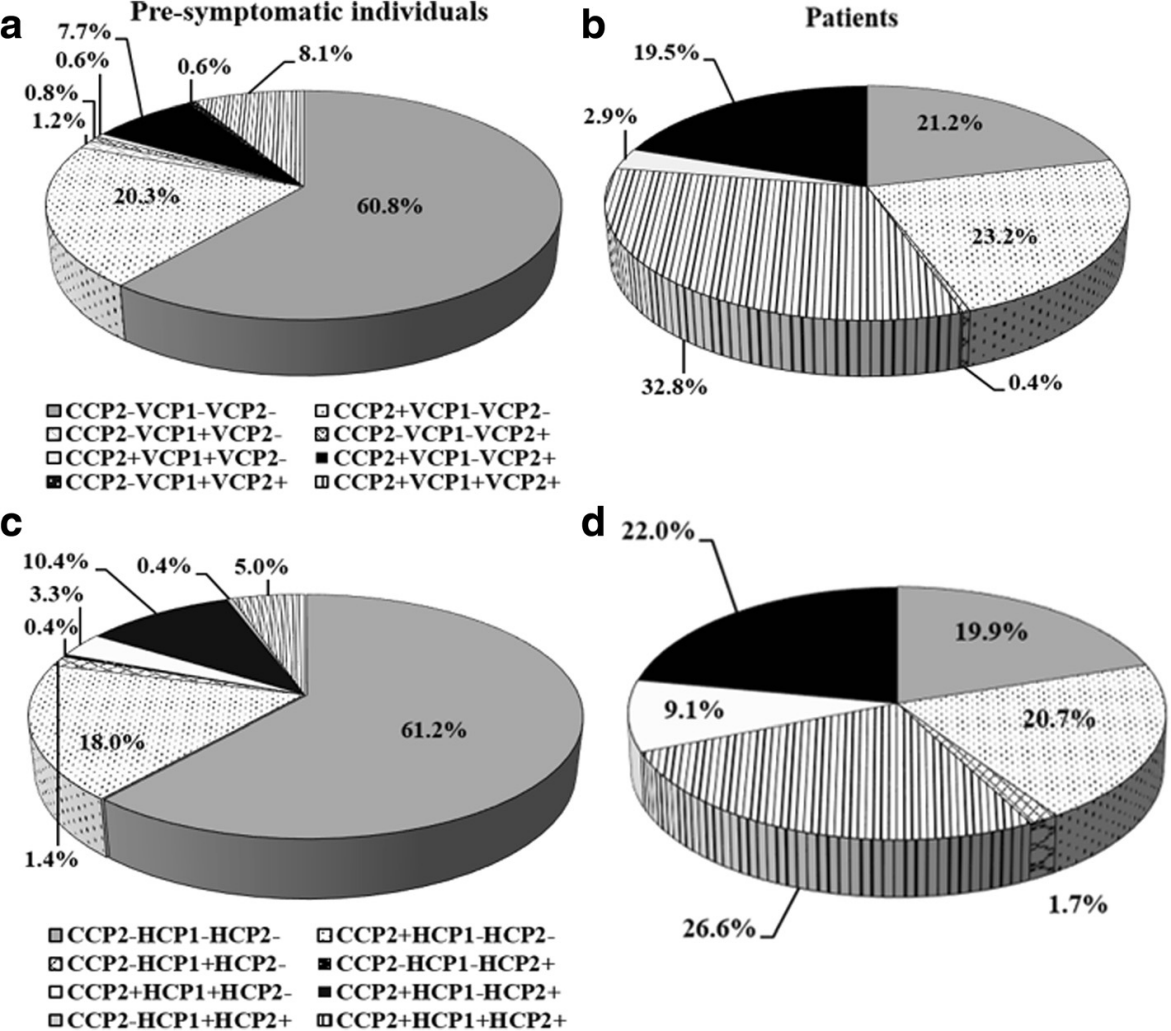
**a**–**d** Clustering of anti-CCP2, anti-VCP1 and anti-VCP2, and anti-CCP2, anti-HCP1 and anti-HCP2 antibodies in individuals before onset of rheumatoid arthritis (RA) symptoms and in patients with RA. *VCP* viral citrullinated peptide derived from Epstein-Barr-virus derived protein, *HCP* histone 4-derived citrullinated peptides, *CCP* cyclic citrullinated peptide

Positivity for each of the four antibodies in the anti-CCP2-positive individuals was almost the same in the pre-symptomatic individuals as the total frequency of positivity for each antibody (anti-VCP1, anti-VCP2, anti-HCP1 and anti-HCP2 antibodies). Only up to 1.7 % of any antibody was detected in anti-CCP2-negative individuals (Additional file 1). Positivity for the three most frequent ACPA (anti-CEP-1, anti-Fibß36-52 and anti-filaggrin antibodies) did not overlap with positivity to the same extent for the four antibodies, anti-VCP1, anti-VCP2, anti-HCP1 and anti-HCP2 (Additional files 2 and 3). In particular, for anti-HCP1 the overlap in pre-symptomatic individuals, was less than 50 % (Additional file 2). Comparison of the distribution of combinations of anti-VCP1 and anti-VCP2 antibodies or anti-HCP1 and anti-HCP2 antibodies, respectively, with anti-CCP2 antibodies in pre-symptomatic individuals and after disease onset revealed different patterns. The frequency of anti-CCP2 positivity increased after disease onset and the combinations of anti-CCP2–, anti-VCP1–, anti-VCP2+ and anti-CCP2–, anti-VCP1+, anti-VCP2+ antibodies disappeared when comparing the pre-symptomatic individuals with patients with RA (Fig. 4a–b). The dominating changes of combinations of anti-CCP2 antibodies with anti-VCP1 and anti-VCP2 or anti-HCP1 and anti-HCP2 when the pre-symptomatic individuals were compared with the patients with RA were that the triple-negative groups (anti-CCP2–, anti-VCP1–, anti-VCP2– or anti-CCP2–, anti-HCP1–, anti-HCP2–, respectively) were reduced from 61.8 % to 21.2 % and 61.2 % to 19.9 %, respectively (Fig. 4c–d). Of the individuals who were triple-negative before the onset of symptoms, approximately 1/3 were still triple-negative after the onset of disease, whilst almost 1/3 became triple-positive, concerning both anti-HCP and anti-VCP antibodies. In all the cases except for those remaining triple-negative the change was created by anti-CCP2 antibodies becoming positive after disease onset in all of the other combinations and thus, moving the individuals to groups including anti-CCP2+. The time pre-dating the onset of symptoms when the samples were collected did not affect the distribution of the combinations of positivity or negativity of the antibodies.

### Anti-VCP1, anti-VCP2, anti-HCP1, and anti-HCP2 IgG in relation to risk factors for disease development and radiological damage

HLA-*DRB1*-SE was associated with anti-VCP2 (*p* < 0.05), and showed a trend for association with anti-HCP2 antibodies (*p* = 0.057) in pre-symptomatic individuals. After disease onset all four antibodies were associated with HLA-SE, (*p* < 0.001–0.063). When analysing the contribution of individual SE alleles in pre-symptomatic individuals, only DRB1*0401 was associated with positivity for anti-VCP2 and for anti-HCP2 antibodies (*p* < 0.05 for both). Pre-symptomatic individuals carrying the HLA-SE and DRB1*0404 allele had significantly higher concentrations of all four antibodies, but was significant only for the DRB1*0404 allele and levels of anti-VCP2 antibodies when adjusted for anti-CCP2 positivity. Individuals with the DRB1*0401 allele had significantly higher levels of anti-VCP1 and anti-VCP2 antibodies compared with DRB1*0401-negative individuals (*p* < 0.001–0.014). The combination of the DRB1*0404 and DRB1*0401 alleles did not increase the risk of developing antibody positivity.

No significant association was found between the four antibodies and age, sex, smoking or the *PTPN22* T-variant in pre-symptomatic individuals (data not shown). In patients with RA being a smoker was only related to the presence of anti-HCP2 antibodies (*p* < 0.05).

The SNPs (n = 314) from the Immunochip encoding for PADI gene loci were analysed in relation to the concentrations of, or positivity for, the four antibodies. In the pre-symptomatic individuals, the concentration of anti-VCP1 antibodies was associated with 57 SNPs, which reduced to 21 when the levels were dichotomized (positive/negative), whilst the other antibodies were only related to a smaller number of the SNPs. None of these relationships remained significant after correcting for multiple testing. The patterns of association was fairly similar in the patients with RA, although with a higher number of associations, and particularly between SNPs and antibody positivity for anti-HCP1 antibodies, where three of the associations remained significant after Bonferroni corrections (rs3003444;imm_1_17485653; *p* < 2.17E-05, p_corr_ < 0.005, rs7542629; imm_1_17495484; *p* < 0.0002, p_corr_ < 0.045, rs11800688; imm_1_17504239; *p* < 6.88E-05, p_corr_ < 0.018). Among the 314 SNPs of ImmunoChip, imm_1_17504969, imm_1_17533086, imm_1_17535391 (corresponding to rs1886303, PADI4_92, PADI4_96) or (rs11203366, rs11203367 and rs874881) of the PADI4 haplotype described by others were not associated with any of the analysed antibodies, either as concentration or positivity, (data not shown) [26-28].

These PADI3/PADI4 SNPs (rs3003444 G-variant, rs7542629 and rs11800688 A-variants) were further analysed and found significantly associated with anti-HCP1 antibody positivity and concentration, independently of adjustments for sex, age, HLA-SE and presence of anti-CCP2 antibodies in patients with RA (Additional file 4).

Positivity for anti-HCP2 antibodies in patients with RA was found to be associated with radiological evidence of increased damage, as detected by higher Larsen scores after 24 months, with a median (Q_1-_Q_3_) score of 10 (4–14) compared with a score of 8 (5–13) in anti-HCP2-negative individuals (*p* < 0.05). This association remained after adjustment for the Larsen score at baseline (*p* = 0.025), but not when adjusting for anti-CCP2 antibodies. No differences could be found between radiological evidence of disease progression and the three other antibodies, anti-VCP1, anti-VCP2 and anti-HCP1 antibodies.

## Discussion

We have recently demonstrated that citrullinated histone-4 from activated neutrophils and viral citrullinated peptides from Epstein-Barr virus nuclear proteins EBNA1 and 2 are targets for ACPA in sera from patients with RA [14, 18, 19]. In this study, we therefore took the opportunity to investigate further the role of citrullinated-histone-4-derived peptides and EBV-derived peptides in the aetiology of RA, by analysing individuals before the onset of symptoms of RA and by comparing the results with those obtained with anti-CCP2 and citrullinated peptides from alpha-enolase, fibrinogenß-chain and filaggrin. Moreover, the association between anti-VCP and anti-HCP antibodies and genes conferring predisposition to RA was also investigated.

The concentrations of the antibodies against the HCP and VCP antibodies were significantly increased in pre-symptomatic individuals and patients with RA, compared with control subjects. The highest frequency (17.1 %) was found for anti-VCP2 followed by anti-HCP2 antibodies (16.3 %) in pre-symptomatic individuals. The frequency in patients with early RA was 52.3 % and 48.5 %, which is consistent with previously published studies [13, 15], even if the frequencies reported in those studies were slightly higher, possibly because of the inclusion of patients with an established disease. The majority of the individuals positive for these four antibodies were also anti-CCP2-positive. Thus, a small percentage of anti-VCP1- and anti- VCP 2-positive and anti-HCP1- and anti-HCP 2-positive individuals were not covered by anti-CCP2 positivity. This evident overlap of anti-CCP2 antibodies with various ACPA is in line with our previous publication [8]. On the contrary, the anti-VCP and anti-HCP antibodies and the three most frequent ACPA, namely antibodies against CEP-1, Fibß36-52 and filaggrin only overlapped to a minor extent, suggesting limited cross-reactivity and/or independent production of these ACPAs.

Positivity for one single antibody confers a low risk of development of RA, which increases when more antibody specificities are present together. In fact, the combination of anti-VCP2 and anti-HCP2 antibodies was found to increase the OR to 17.2, but the highest ORs were for anti-VCP1, anti-VCP2 and anti-HCP1, together with anti-CCP2 antibodies. The OR for anti-CCP2 antibodies alone was lower in the pre-symptomatic individuals (OR = 21.9). In our previous study the OR was higher for the combination of two ACPA (e.g., anti-CEP1 and anti-Fib36-52 antibodies) (OR = 40.9, 95 % CI 19.8, 82.3), although the number of analysed samples from pre-symptomatic individuals and controls were much higher, thus making comparisons on exact values difficult [8]. The major changes that occurred in the distribution of the combinations of anti-CCP2 and anti-VCP1 or anti-VCP2 or anti-HCP1 or anti-HCP2 positivity comparing the pre-symptomatic individuals with the patients after diagnosis was that triple antibody negativity converted to triple antibody positivity. This conversion confirmed that both anti-HCP and anti-VCP antibodies appear to play a potential role in the development of RA, e.g., as they display a “classical” ACPA pattern [8]. During the period pre-dating the onset of symptoms the number of positive antibodies increased the closer to onset of symptoms the samples were collected, thus, suggesting an epitope spreading, as already reported in other studies [8, 29]. From this study, we did not detect any time difference in the appearance of positivity for any of the antibodies against the citrullinated proteins from histone H4 or from the EBV-derived proteins compared with the other ACPA or anti-CCP2 antibodies. Thus, examining the time course of antibody appearance, no hierarchy among citrullinated epitopes of endogenous or exogenous origin could be identified and no clues about a single inciting antigen were obtained.

Examining the influence of genes conferring susceptibility to RA, all four antibodies were found to be associated with HLA-SE after disease onset; on the contrary, only anti-VCP2 antibodies were associated with HLA-SE, even before symptom onset, as was already observed with other ACPA specificities [30]. When analysing the contribution of individual SE alleles in pre-symptomatic individuals, the *0401 allele was associated with both anti-VCP2 and anti-HCP2 antibody positivity, in line with previous findings in a French Caucasian population [31, 32]. Among the other genetic factors analysed, we found no association between anti-VCP or anti-HCP and *PTPN22*. However, three of the SNPs (rs3003444, rs7542629 and rs11800688) in the *PADI3/PADI4* intergenic region remained significantly associated with anti-HCP1 antibodies after Bonferroni corrections and after adjustments for e.g., anti-CCP2 antibodies, HLA-SE, age and sex. These data suggest a relationship between PADI polymorphisms and histone citrullination, a mechanism hypothesised to be essential for the formation of NETs and thereby the release of RA-associated autoantigens. The SNP (rs3003444, A-allele) of *PADI3/PADI4* has been shown to be associated with disease development in ACPA-positive patients with RA, who are of Chinese origin [33], but no data are yet available in Caucasian patients. The concentrations of anti-CCP2 and anti-MCV antibodies have been associated with a haplotype comprising three SNPs within the PADI4 gene [27, 28]. These SNPs were not associated with the antibodies analysed in this study, including anti-CCP2 antibodies.

Irrespective of the potential inciting antigen of exogenous or endogenous origin, ACPA specific for VCP and HCP during the pre-symptomatic period follow the same pattern (time of appearance and increase in concentrations before onset). The present study suggests that these antibodies have a potential role in the pathogenesis of RA that could not be completely explained by, e.g., anti-CEP-1, anti-Fibß36-52 or anti-filaggrin antibodies alone and that certain combinations of the anti-VCP1, anti-VCP2, anti-HCP1 and anti-HCP2 antibodies are associated with an increased risk of developing RA. These results suggest the need for investigating the relationship between EBV infection and anti-VCP/ACPA production in longitudinal studies involving patients pre RA. The described association between the *PADI3/PADI4* polymorphism and anti-HCP1 antibodies suggests a novel link between deimination and production of ACPA. Our study has limitations: the samples analysed were from different population surveys, and were not collected on a regular basis. However, we believe this to be the largest population-based study performed to date analysing anti-VCP and anti-HCP antibody responses in individuals before onset of the symptoms of RA. Further studies are also needed to more deeply investigate, in RA as well as in other autoimmune disorders where NETosis is involved, the role of *PADI3/PADI4* SNPs in affecting enzyme levels and/or activity and possibly histone deimination and NET formation.

## Conclusions

Anti-VCP and anti-HCP antibodies pre-date symptom onset and predict the development of RA, without hierarchy among citrullinated epitopes. These results suggest that no inciting citrullinated antigen is common to all patients with RA. The association between the PADI3/PADI4 polymorphism and anti-HCP1 antibodies suggests a link between deimination and ACPA.

## Abbreviations

ACPA, anti-citrullinated peptide antibodies; ACR, American College of Rheumatology; Anti-CCP, anti-cyclic citrullinated peptide; Anti-CEP1, antibodies against α-enolase; Anti-Fibß36-52, antibodies against Fibrinogenβ36-52; anti-HCP1/HCP2, antibodies against histone 4-derived citrullinated peptides; anti-VCP1/VCP2, antibodies against viral citrullinated peptides derived from Epstein-Barr-virus encoded protein; AU, arbitrary units; CI, confidence interval; EBNA1/EBNA2, viral citrullinated peptides derived from Epstein-Barr-virus encoded protein; EBV, Epstein-Barr virus; ELISA, enzyme-linked immunosorbent assay; ELS, ectopic lymphoid structures; EULAR, European League Against Rheumatism; HLA-SE, human leukocyte antigen – shared epitope; IQR, interquartile range; NETS, neutrophil extracellular traps; OR, odds ratio; PAD, peptidylarginine deiminase; PTPN22, protein tyrosine phosphatase, non-receptor type 22; RA, rheumatoid arthritis; ROC, receiver operating characteristic; *r*_s_, Spearman’s rank correlation coefficient; SNP, single nucleotide polymorphism

## Additional files

Additional file 1: Figure S1. A) Anti-CCP2, anti-VCP1 and anti-VCP2, and B) anti-CCP2, anti-HCP1 and anti-HCP2 antibodies and their combinations of positivity, in pre-symptomatic individuals. Results are presented as number (%) of positive individuals. (PDF 49 kb) Additional file 2: Figure S2. A) Anti-CCP1, anti-HCP1 anti-HCP2, B) anti-Fibβ36-52, anti-HCP1 and anti-HCP2 and C) anti-CEP1, anti-HCP1, anti-HCP2 antibodies and their combinations of positivity, in pre-symptomatic individuals. Results are presented as number (%) of positive individuals. (PDF 61 kb) Additional file 3: Figure S3. A) anti-CCP1, anti-VCP1 and anti-VCP2, B) anti-Fibβ36-52, anti-VCP1 and anti-VCP2 and C) anti-CEP1, anti-VCP1 and anti-VCP2 antibodies and their combinations of positivity, in pre-symptomatic individuals. Results are presented as number (%) of positive individuals. (PDF 67 kb) Additional file 4: Table S1. Significant single nucleotide polymorphism (SNP) variants associated with anti-HCP1 positivity in patients with RA. (PDF 75 kb)

## References

[1] Klareskog L, Padyukov L, Lorentzen J, Alfredsson L (2006). Mechanisms of disease: Genetic susceptibility and environmental triggers in the development of rheumatoid arthritis. Nat Clin Pract Rheumatol..

[2] Anzilotti C, Pratesi F, Tommasi C, Migliorini P (2010). Peptidylarginine deiminase 4 and citrullination in health and disease. Autoimmun Rev..

[3] Pruijn GJ, Wiik A, van Venrooij WJ (2010). The use of citrullinated peptides and proteins for the diagnosis of rheumatoid arthritis. Arthritis Res Ther..

[4] Uysal H, Nandakumar KS, Kessel C (2010). Antibodies to citrullinated proteins: molecular interactions and arthritogenicity. Immunol Rev..

[5] Aletaha D, Neogi T, Silman AJ (2010). 2010 rheumatoid arthritis classification criteria: an American College of Rheumatology/European League Against Rheumatism collaborative initiative. Ann Rheum Dis..

[6] Rantapää-Dahlqvist S, de Jong BAW, Berglin E (2003). Antibodies against cyclic citrullinated peptide and IgA rheumatoid factor predict the development of rheumatoid arthritis. Arthritis Rheum..

[7] Nielen MMJ, van Schaardenburg D, Reesink HW (2004). Specific autoantibodies precede the symptoms of rheumatoid arthritis: a study of serial measurements in blood donors. Arthritis Rheum..

[8] Brink M, Hansson M, Mathsson L (2013). Multiplex analyses of antibodies against citrullinated peptides in individuals prior to development of rheumatoid arthritis. Arthritis Rheum..

[9] Suwannalai P, van de Stadt LA, Radner H (2012). Avidity maturation of anti-citrullinated protein antibodies in rheumatoid arthritis. Arthritis Rheum..

[10] van der Woude D, Alemayehu WG, Verduijn W (2010). Gene-environment interaction influences the reactivity of autoantibodies to citrullinated antigens in rheumatoid arthritis. Nat Genet..

[11] Croia C, Serafini B, Bombardieri M (2013). Epstein-Barr virus persistence and infection of autoreactive plasma cells in synovial lymphoid structures in rheumatoid arthritis. Ann Rheum Dis..

[12] Toussirot E, Roudier J (2007). Pathophysiological links between rheumatoid arthritis and the Epstein-Barr virus: an update. Joint Bone Spine..

[13] Dwivedi N, Upadhyay J, Neeli I (2012). Felty’s syndrome autoantibodies bind to deiminated histones and neutrophil extracellular chromatin traps. Arthritis Rheum..

[14] Pratesi F, Dioni I, Tommasi C (2014). Antibodies from patients with rheumatoid arthritis target citrullinated histone 4 contained in neutrophils extracellular traps. Ann Rheum Dis..

[15] Neumann A, Berends ETM, Nerlich A (2014). The antimicrobial peptide LL-37 facilitates the formation of neutrophil extracellular traps. Biochem J..

[16] Khandpur R, Carmona-Rivera C, Vivekanandan-Giri A, et al. NETs are a source of citrullinated autoantigens and stimulate inflammatory responses in rheumatoid arthritis. Sci Transl Med. 2013;5:178ra40. 10.1126/scitranslmed.3005580 PMC3727661 23536012

[17] Corsiero E, Bombardieri M, Carlotti E (2015). Single cell cloning and recombinant monoclonal antibodies generation from RA synovial B cells reveal frequent targeting of citrullinated histones of NETs. Ann Rheum Dis.

[18] Pratesi F, Tommasi C, Anzilotti C, Chimenti D, Migliorini P (2006). Deiminated Epstein-Barr virus nuclear antigen 1 is a target of anti-citrullinated protein antibodies in rheumatoid arthritis. Arthritis Rheum..

[19] Pratesi F, Tommasi C, Anzilotti C (2011). Antibodies to a new viral citrullinated peptide, VCP2: fine specificity and correlation with anti-cyclic citrullinated peptide (CCP) and anti-VCP1 antibodies. Clin Exp Immunol..

[20] Arnett FC, Edworthy SM, Bloch DA (1988). The American Rheumatism Association 1987 revised criteria for the classification of rheumatoid arthritis. Arthritis Rheum..

[21] Berglin E, Johansson T, Sundin U (2006). Radiological outcome in rheumatoid arthritis is predicted by presence of antibodies against cyclic citrullinated peptide before and at disease onset, and by IgA-RF at disease onset. Ann Rheum Dis..

[22] Bruynesteyn K, Boers M, Kostense P, van der Linden S, and van der Heijde D. Deciding on progression of joint damage in paired films of individual patients: smallest detectable difference or change. Ann Rheum Dis. 2005;64:179-82. 10.1136/ard.2003.018457 PMC1755378 15286006

[23] Kokkonen H, Johansson M, Innala L, Jidell E, Rantapaa-Dahlqvist S (2007). The PTPN22 1858C/T polymorphism is associated with anti-cyclic citrullinated peptide antibody-positive early rheumatoid arthritis in northern Sweden. Arthritis Res Ther..

[24] Eyre S, Bowes J, Diogo D (2012). High-density genetic mapping identifies new susceptibility loci for rheumatoid arthritis. Nat Genet..

[25] Purcell S, Neale B, Todd-Brown K (2007). PLINK: a tool set for whole-genome association and population-based linkage analyses. Am J Hum Genet..

[26] Guzmán-Guzmán IP, Reyes-Castillo Z, Muñoz-Barrios S (2015). Polymorphisms and functional haplotype in PADI4: further evidence for contribution on rheumatoid arthritis susceptibility and anti-cyclic citrullinated peptide antibodies in a western Mexican population. Immunol Lett..

[27] Reyes-Castillo Z, Palafox-Sánchez CA, Parra-Rojas I (2015). Comparative analysis of autoantibodies targeting peptidylarginine deiminase type 4, mutated citrullinated vimentin and cyclic citrullinated peptides in rheumatoid arthritis: associations with cytokine profiles, clinical and genetic features. Clin Exp Immunol..

[28] Caponi L, Petit-Teixeira E, Sebbag M (2005). A family based study shows no association between rheumatoid arthritis and the PADI4 gene in a white French population. Ann Rheum Dis..

[29] van der Woude D, Rantapaa-Dahlqvist S, Ioan-Facsinay A (2010). Epitope spreading of the anti-citrullinated protein antibody response occurs before disease onset and is associated with the disease course of early arthritis. Ann Rheum Dis..

[30] Kokkonen H, Brink M, Hansson M (2015). Associations of antibodies against citrullinated peptides with human leukocyte antigen-shared epitope and smoking prior to the development of rheumatoid arthritis. Arthritis Res Ther..

[31] Saal JG, Krimmel M, Steidle M (1999). Synovial Epstein-Barr virus infection increases the risk of rheumatoid arthritis in individuals with the shared HLA-DR4 epitope. Arthritis Rheum..

[32] Pratesi F, Petit-Teixeira E, Sidney J (2012). Effect of rheumatoid arthritis (RA) susceptibility genes on the immune response to viral citrullinated peptides in RA. J Rheumatol..

[33] Too CL, Murad S, Dhaliwal JS (2012). Polymorphisms in peptidylarginine deiminase associate with rheumatoid arthritis in diverse Asian populations: evidence from MyEIRA study and meta-analysis. Arthritis Res Ther..

