# Supplementary figures and images for: Antibodies directed against endogenous and exogenous citrullinated antigens pre-date the onset of rheumatoid arthritis
Source: Arthritis Res Ther. 2016 Jun 3;18:127. doi: 10.1186/s13075-016-1031-0 (PMC4891920; doi:10.1186/s13075-016-1031-0)

## Supplementary Figure 1

a)

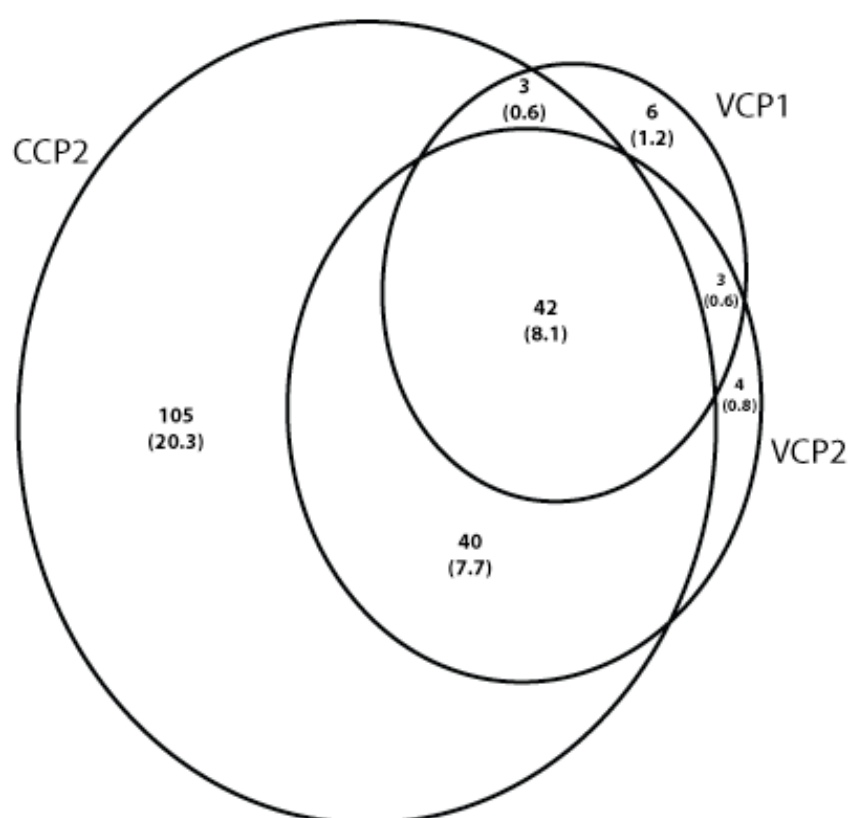

b)

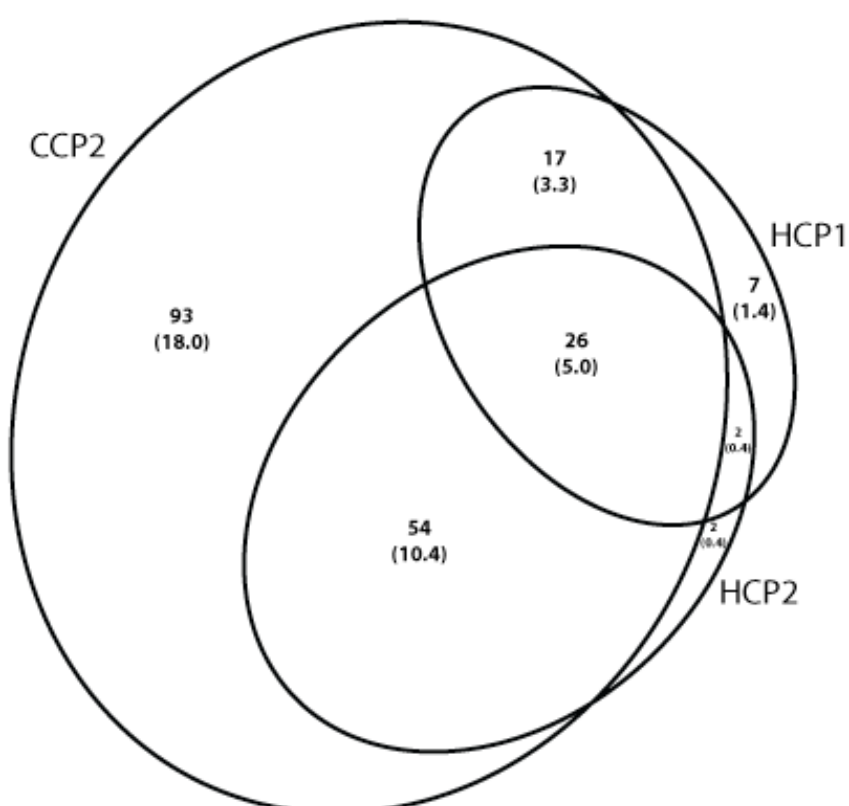

Supplement: Additional file 1: Figure S1. — A) Anti-CCP2, anti-VCP1 and anti-VCP2, and B) anti-CCP2, anti-HCP1 and anti-HCP2 antibodies and their combinations of positivity, in pre-symptomatic individuals. Results are presented as number (%) of positive individuals. (PDF 49 kb) [file 13075_2016_1031_MOESM1_ESM.pdf]

Supplementary Figure 2

a)

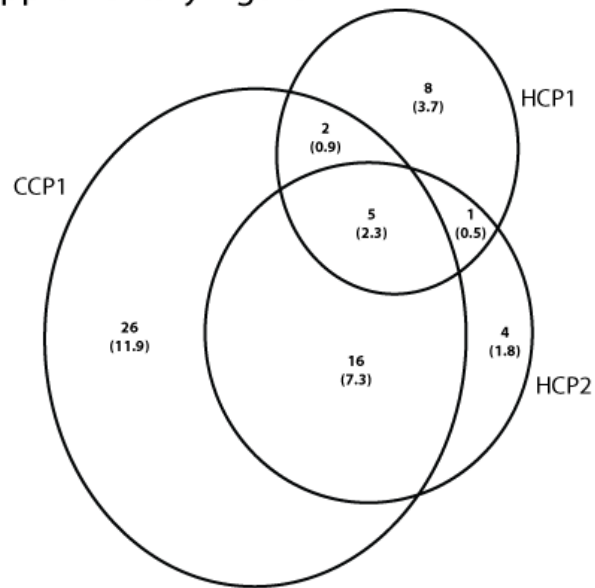

b)

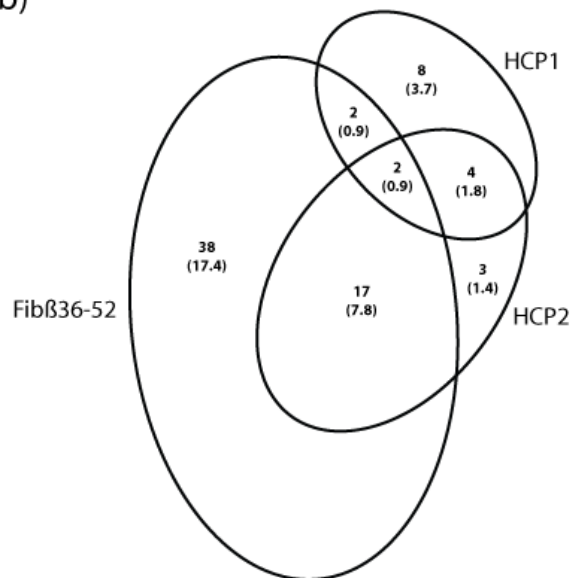

c)

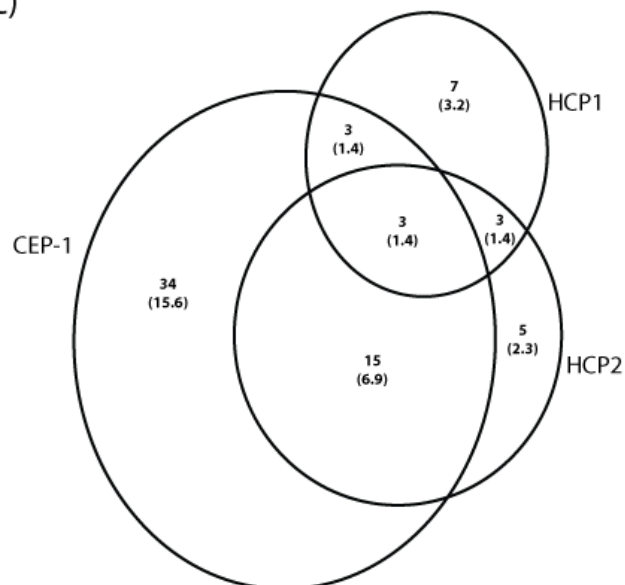

Supplement: Additional file 2: Figure S2. — A) Anti-CCP1, anti-HCP1 anti-HCP2, B) anti-Fibβ36-52, anti-HCP1 and anti-HCP2 and C) anti-CEP1, anti-HCP1, anti-HCP2 antibodies and their combinations of positivity, in pre-symptomatic individuals. Results are presented as number (%) of positive individuals. (PDF 61 kb) [file 13075_2016_1031_MOESM2_ESM.pdf]

Supplementary Figure 3

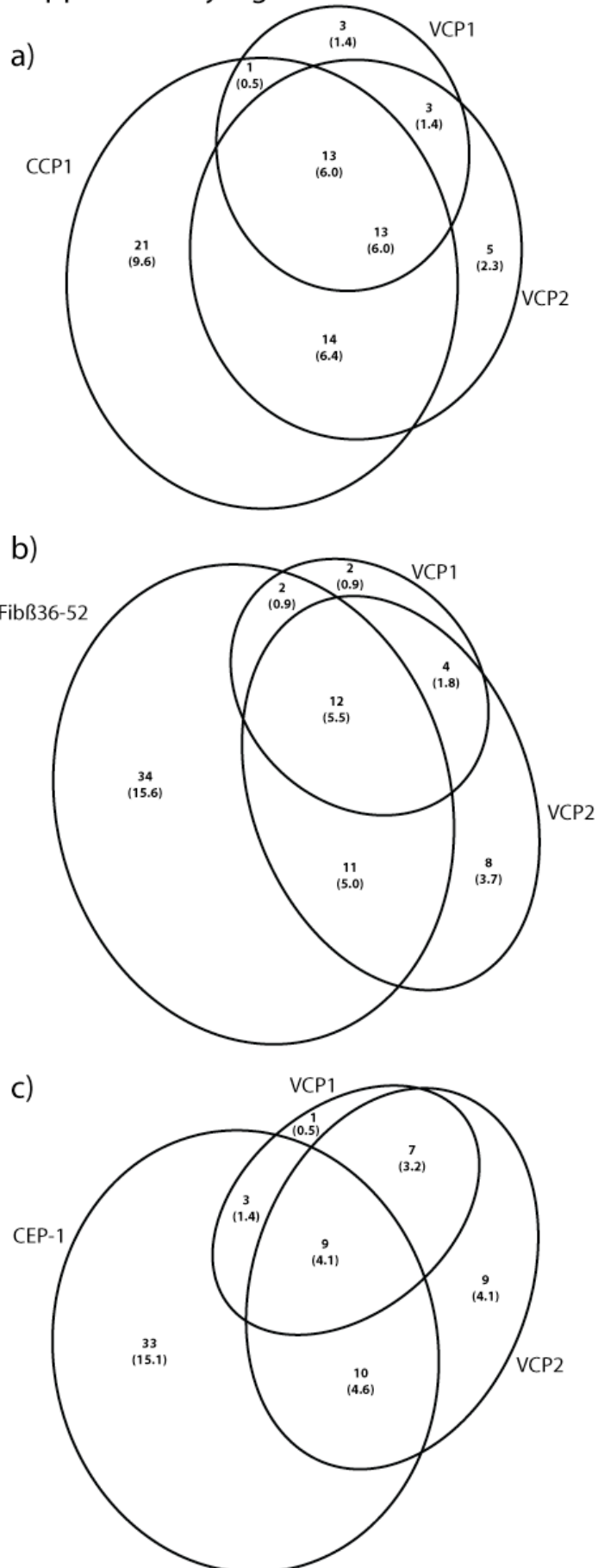

Supplement: Additional file 3: Figure S3. — A) anti-CCP1, anti-VCP1 and anti-VCP2, B) anti-Fibβ36-52, anti-VCP1 and anti-VCP2 and C) anti-CEP1, anti-VCP1 and anti-VCP2 antibodies and their combinations of positivity, in pre-symptomatic individuals. Results are presented as number (%) of positive individuals. (PDF 67 kb) [file 13075_2016_1031_MOESM3_ESM.pdf]
