# Supplementary material for: Antibodies directed against endogenous and exogenous citrullinated antigens pre-date the onset of rheumatoid arthritis
Source: Arthritis Res Ther. 2016 Jun 3;18:127. doi: 10.1186/s13075-016-1031-0 (PMC4891920; doi:10.1186/s13075-016-1031-0)
Supplement: Additional file 4: Table S1. — Significant single nucleotide polymorphism (SNP) variants associated with anti-HCP1 positivity in patients with RA. (PDF 75 kb) [file 13075_2016_1031_MOESM4_ESM.pdf]

Supplementary table 1. Significant SNP variants associated with anti-HCP1 positivity in RA patients

**PADI3/PADI4** *SNP variant*	**Anti-HCP1 antibody positivity**					**Anti-HCP1 antibody concentration**			
	**Risk allele**	**Unadjusted** OR 95%Cl[2]	**P-value**	**Adjusted**[1] OR 95%Cl[2]	**P-value**	**Unadjusted** OR 95%Cl[2]	**P -value**	**Adjusted**[1] OR 95%Cl[2]	**P- value**
rs3003444	**G**	3.4 (1.8, 6.4)	<0.001	3.3 (1.6, 6.6)	0.001	38.5 (29.4, 47,5)	<0.001	48.0 (8.0, 88.1)	0.019
rs7542629	**A**	2.4 (1.3, 4.3)	0.005	2.1 (1.2, 4.2)	0.026	40.0 (29.4, 50.6)	<0.001	50.7 (9.5, 91.8)	0.016
rs11800688	**A**	2.7 (1.4, 5.1)	0.002	3.0 (1.4, 6.1)	0.003	38.7 (28.9, 58.6)	<0.001	61.0 (25.0,97.0)	0.001

[1]Adjustments made for age, sex, carriage of HLA-SE and presence of anti-CCP2 antibody. [2]Odds ratio (OR) 95 % confidence interval (Cl)
